# Cellular and Molecular Features of Developmentally Programmed Genome Rearrangement in a Vertebrate (Sea Lamprey: *Petromyzon marinus*)

**DOI:** 10.1371/journal.pgen.1006103

**Published:** 2016-06-24

**Authors:** Vladimir A. Timoshevskiy, Joseph R. Herdy, Melissa C. Keinath, Jeramiah J. Smith

**Affiliations:** 1 Department of Biology, University of Kentucky, Lexington, Kentucky, United States of America; 2 Laboratory of Genetics, The Salk Institute, La Jolla, California, United States of America; Oregon Health and Science University, UNITED STATES

## Abstract

The sea lamprey (*Petromyzon marinus*) represents one of the few vertebrate species known to undergo large-scale programmatic elimination of genomic DNA over the course of its normal development. Programmed genome rearrangements (PGRs) result in the reproducible loss of ~20% of the genome from somatic cell lineages during early embryogenesis. Studies of PGR hold the potential to provide novel insights related to the maintenance of genome stability during the cell cycle and coordination between mechanisms responsible for the accurate distribution of chromosomes into daughter cells, yet little is known regarding the mechanistic basis or cellular context of PGR in this or any other vertebrate lineage. Here we identify epigenetic silencing events that are associated with the programmed elimination of DNA and describe the spatiotemporal dynamics of PGR during lamprey embryogenesis. *In situ* analyses reveal that the earliest DNA methylation (and to some extent H3K9 trimethylation) events are limited to specific extranuclear structures (micronuclei) containing eliminated DNA. During early embryogenesis a majority of micronuclei (~60%) show strong enrichment for repressive chromatin modifications (H3K9me3 and 5meC). These analyses also led to the discovery that eliminated DNA is packaged into chromatin that does not migrate with somatically retained chromosomes during anaphase, a condition that is superficially similar to lagging chromosomes observed in some cancer subtypes. Closer examination of “lagging” chromatin revealed distributions of repetitive elements, cytoskeletal contacts and chromatin contacts that provide new insights into the cellular mechanisms underlying the programmed loss of these segments. Our analyses provide additional perspective on the cellular and molecular context of PGR, identify new structures associated with elimination of DNA and reveal that PGR is completed over the course of several successive cell divisions.

## Author Summary

Lampreys possess a fascinating genome biology wherein large portions of the genome, including large numbers of genes, are programmatically deleted during development. The lamprey therefore represents a uniquely informative system with respect to several broad areas of biology, including genome stability/rearrangement, epigenetic silencing, and the establishment and maintenance of pluripotency. However, little is known regarding the cellular context or mechanism of deletion, partly due to the challenges of observing rearrangements *in situ*. Here we present analyses and new techniques that significantly advance our understanding of the subcellular context of programmed rearrangements and interactions between programmed deletion and canonical DNA silencing mechanisms. These analyses demonstrate that DNA elimination occurs earlier in embryogenesis than was previously recognized and reveal several new cellular and molecular aspects of programmed DNA loss. Specifically we show that eliminated DNA exhibits a unique migration pattern during cell division, is packaged into discreet subcellular structures later in the cell cycle, and undergoes epigenetic silencing through DNA and histone methylation. These observations provide new insight into the mechanisms underlying programmed DNA loss and suggest a functional link between programmed DNA loss and other, more conserved gene silencing pathways.

## Introduction

The sea lamprey (*Petromyzon marinus*) represents one of the few vertebrate species known to undergo large-scale programmatic elimination of genomic DNA over the course of its normal development [1–4]. Programmed genome rearrangements (PGRs) result in the reproducible loss of ~20% of the genome from somatic cell lineages and a reduction of chromosome number from ~198 to ~164 (2N) [4–6]. Previous studies have shown that DNA is physically eliminated during the transition between gastrula and blastula stages: between the second and third day of development [4]. Given that most aspects of lamprey’s developmental and cellular biology are conserved with other vertebrates [7–10], PGR holds the potential to provide novel insights related to maintenance of genome stability and interactions between various cellular mechanisms responsible for the proper segregation of chromosomes.

Lampreys are by no means the only organisms that undergo large-scale programmed rearrangement of their genomes. Organisms known to undergo PGR include diverse protozoan, invertebrate and vertebrate taxa, and the mechanisms underlying PGR are thought to be similarly diverse [11–19]. Studies of these independent acquisitions have revealed common themes that speak to the underlying logic of PGR and its integration with other epigenetic silencing pathways [11–14]. In many taxa PGR is known to occur early in development and results in the targeted elimination of specific genomic segments from essentially all somatic cell lineages, with targeted segments being retained exclusively by the germline. Studies in lamprey and the nematode *Ascaris suum* have shown that eliminated DNA encodes genes that are expressed in mature gonads and embryonic germ cells [6, 20], supporting the interpretation that PGR likely serves as an irreversible mechanism of silencing genes within somatic cell lineages.

Studies performed on diverse taxa suggest that PGR-mediated silencing may often interact cooperatively with other silencing pathways. In the ciliates both DNA methylation/hydroxymethylation and methylation of histone H3 at lysine 9 (H3K9me) are associated with programmed elimination [11, 21]. In sciarid flies embryonic elimination of the paternal X chromosome is associated with retention of H3S10 hyperphosphorylation (H3S10P) during late anaphase, which may contribute to silencing by preventing decondensation and access to H3K9 by methyltransferases [12, 13]. Similarly, in zebra finch a single germline-restricted chromosome is heavily marked by both trimethylated H3K9 (H3K9me3) and acetylated H4K16 in meiotic testes (the chromosome is eliminated at the end of male meiosis and only transmitted by oocytes, although embryonic elimination has not been directly observed) [14].

Little is known regarding the mechanistic basis or cellular context of PGR in any vertebrate lineage. Given the high fecundity of lampreys and the fact that fertilization and all stages of embryonic development occur externally, lamprey provides a powerful system for observing and manipulating cells during the process of PGR. Here we describe epigenetic correlates of PGR and the spatiotemporal dynamics of DNA elimination in lamprey. *In situ* analyses revealed that the earliest DNA methylation events target specific extranuclear structures (micronuclei) that contain DNA eliminated by PGR. The spatiotemporal resolution of these analyses also permitted the discovery of other reproducible subcellular features that are associated with the differential segregation of retained vs. eliminated DNA and the packaging of eliminated DNA into micronuclei. Specifically, eliminated DNA appears to be packaged into chromatin that does not migrate with somatically retained chromosomes and is superficially similar to lagging chromosomes that are observed in some cancer subtypes [22–24]. Closer examination of “lagging” chromatin reveals distributions of repetitive elements, cytoskeletal contacts and apparent chromatin contacts that provide new insights into the cellular mechanisms underlying the programmed loss of these segments.

## Results and Discussion

### Repressive Chromatin and Extranuclear DNA in Rearranging Embryos

**Fig 1:**
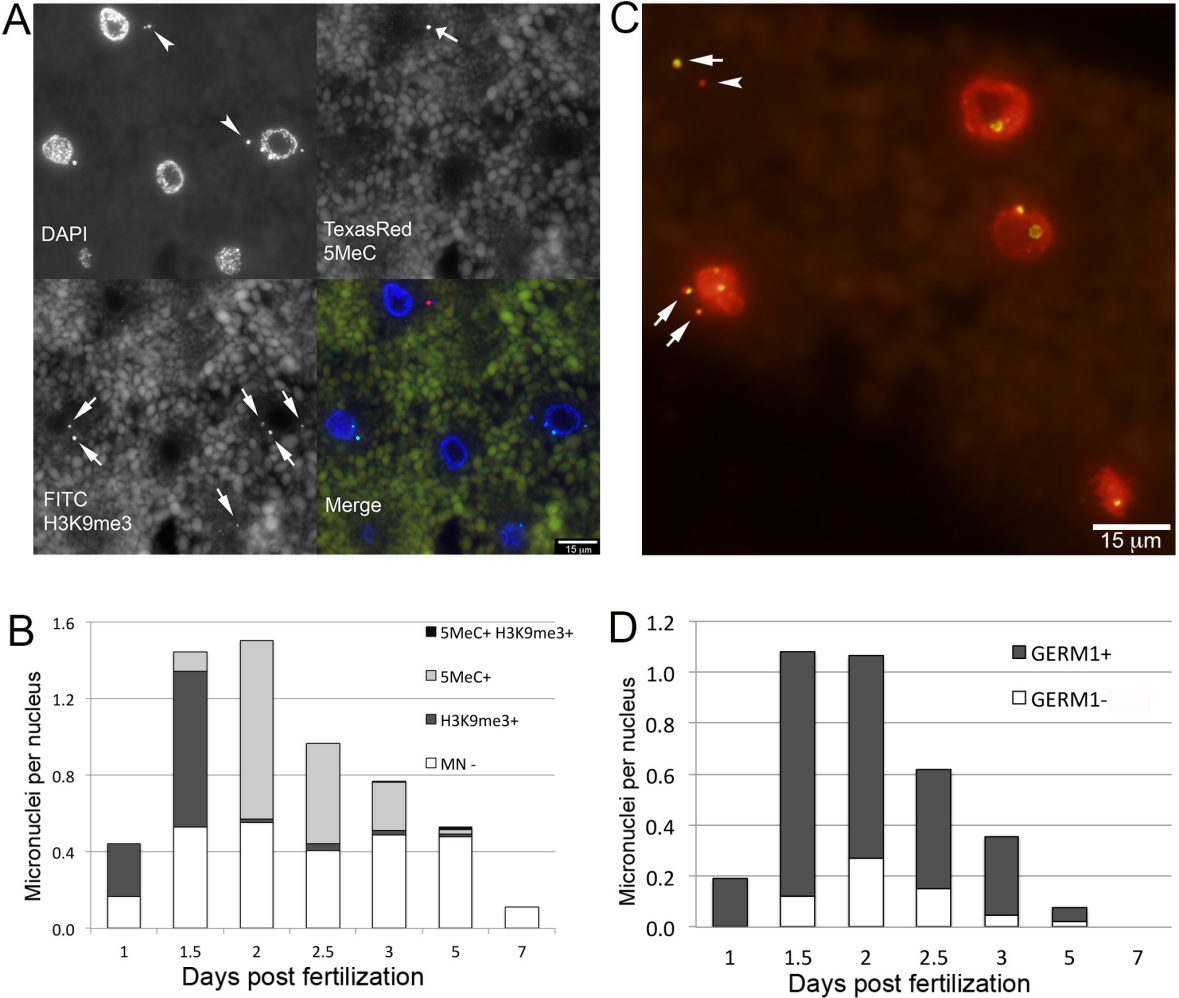
Micronuclei are abundant in embryos undergoing programmed genome rearrangement. (A) 5-Methylcytosine (5MeC) and trimethyl-H3K9 (H3K9me3) immunolabeling of embryos at 1.5 dpf. (B) Distributions of 5MeC and H3K9me3 in micronuclei during early embryogenesis. (C) Fluorescence *in situ* hybridization of the *Germ1* probe (green) to embryos at 1.5 dpf, true color image of nuclei counterstained with propidium iodide. (D) Distributions of *Germ1*-positive and *Germ1*-negative micronuclei in early embryonic stages. Arrows and arrowheads mark signal-positive and signal-negative micronuclei, respectively.

Programmed DNA elimination is sparsely distributed across the tree of life and likely arose several times over metazoan evolution [19] yet in several species programmed elimination of DNA has been shown to act cooperatively with other, more conserved, epigenetic silencing pathways [11–14]. To investigate possible interactions between PGR and early gene silencing events, we applied indirect immunofluorescence labeling using antibodies against 5-methylcytosine (5meC), histone 3 trimethylated at lysine 9 (H3K9me3) and histone 3 trimethylated at lysine 27 (H3K27me3) to characterize the distribution of these modifications during early embryogenesis. In general these repressive modifications were essentially absent at the earliest developmental time points and increased in abundance during the first week of development. Similar patterns have been observed for several vertebrate and invertebrate species, reflecting reprogramming events that are involved in the initial establishment of pluripotency following fertilization (i.e. global demethylation) and the subsequent onset of zygotic genome activation [25, 26]. However, the subcellular localization of two modifications (5meC and H3K9me3) deviated from the typical pattern that has been described for other taxa. During the first two days of development, days post fertilization (dpf) 5meC and H3K9me3 immunofluorescence localized almost exclusively to DAPI-positive extranuclear structures (micronuclei–MNi, Fig 1A). To more thoroughly test whether micronuclei are associated with the elimination of DNA via PGR, we performed *in situ* hybridization with the germline-enriched repetitive element *Germ1*. This sequence is highly abundant within the germline and only localizes to two somatically retained chromosomes [6]. These analyses revealed that a majority of MNi, though not all, contain the *Germ1* repeat, consistent with the interpretation that these micronuclei contain material destined for elimination from somatic lineages via PGR (Fig 1C and 1D, S1 Table).

Two-color immunolabeling of 5MeC and H3K9me3 revealed that these heterochromatic marks occur in largely non-overlapping sets of MNi and vary in prevalence over the first several days of embryogenesis (Fig 1A and 1B, S2 Table). At 1 dpf, 5MeC was essentially absent from both nuclei and micronuclei, whereas ~60% of micronuclei showed strong immunolabeling for H3K9me3. At 1.5 dpf the proportion of H3K9me3 positive MNi remained relatively stable, and the first 5MeC positive MNi were observed, albeit at a relatively low frequency (~7% of MNi). At 2 dpf the proportion of 5MeC positive MNi significantly increased and the proportion of H3K9me3 positive MNi significantly decreased, with localization of H3K9me3 transitioning to the primary nucleus (Fig 1A, S1D Fig, S2 Table). Similar patterns of 5MeC and H3K9me3 immunolabeling were observed at 2.5 and 3 dpf. Coordinate with changes in the distribution of epigenetic modifications, the abundance of MNi also changed dynamically over the first week of embryogenesis, rising sharply at 1.5 dpf, peaking at 2dpf and approaching zero by 7 dpf (Fig 1B and 1D).

The developmental profile and subcellular localization of H3K9me3 and 5MeC marks suggest that these epigenetic modifications may mark MNi in different phases of elimination. Micronuclei with elevated levels of H3K9me3 were predominantly located in close proximity to the primary nucleus, whereas MNi with elevated levels of 5MeC were typically located at more distal sites (Fig 1A, S1C and S1D Fig). The timing and location of MNi with chromatin repressive marks suggest that H3K9 tri-methylation marks recently formed MNi and that 5MeC may mark older MNi. It seems plausible that DNA methylation might act to ensure transcriptional silencing of material in MNi prior to its complete elimination. Notably, similar interactions between H3K9 and DNA methylation have been observed during heterochromatin formation and chromatin-remodeling in organisms that do not undergo PGR (fungi [27], plants [28], and mammals [29]).

In comparison to 5MeC, the repressive histone mark H3K9me3 showed a somewhat more complex pattern over the course of the cell cycle. This mark localizes to condensed chromosomes during metaphase and persists through telophase/cytokinesis but is essentially absent from interphase nuclei (S1A–S1C Fig, Fig 1A). The presence of H3K9me3 in newly formed micronuclei suggests that micronuclear H3K9me3 marks are remodeled more slowly than their primary nuclear counterparts following M phase. Cell cycle-dependent changes in histone H3 methylation have been reported for mammalian systems, which do not undergo PGR, and appear to be necessary for proper mitotic segregation [30–32]. Moreover, studies in both mammalian and non-mammalian systems have shown that H3K9 methylation is critical for anchoring heterochromatin to the nuclear envelope [33]. Immunolabeling of nuclear envelope markers lamin B1, and nuclear pore o-linked glycoprotein in rearranging embryos reveals that both of these proteins localize to interphase nuclei, but are absent from micronuclei (S2A and S2B Fig). In human, depletion of LMN-B1 and pore complex proteins are associated with nuclear membrane defects in the context of cancer [34]. Taken together, these studies indicate that retention of H3K9me3 in newly formed MNi might play functional roles in maintaining chromatin compaction, positioning eliminated chromatin, or recruiting other structural components of MNi.

### Segregation of Retained Chromosomes and Eliminated DNA

**Fig 2:**
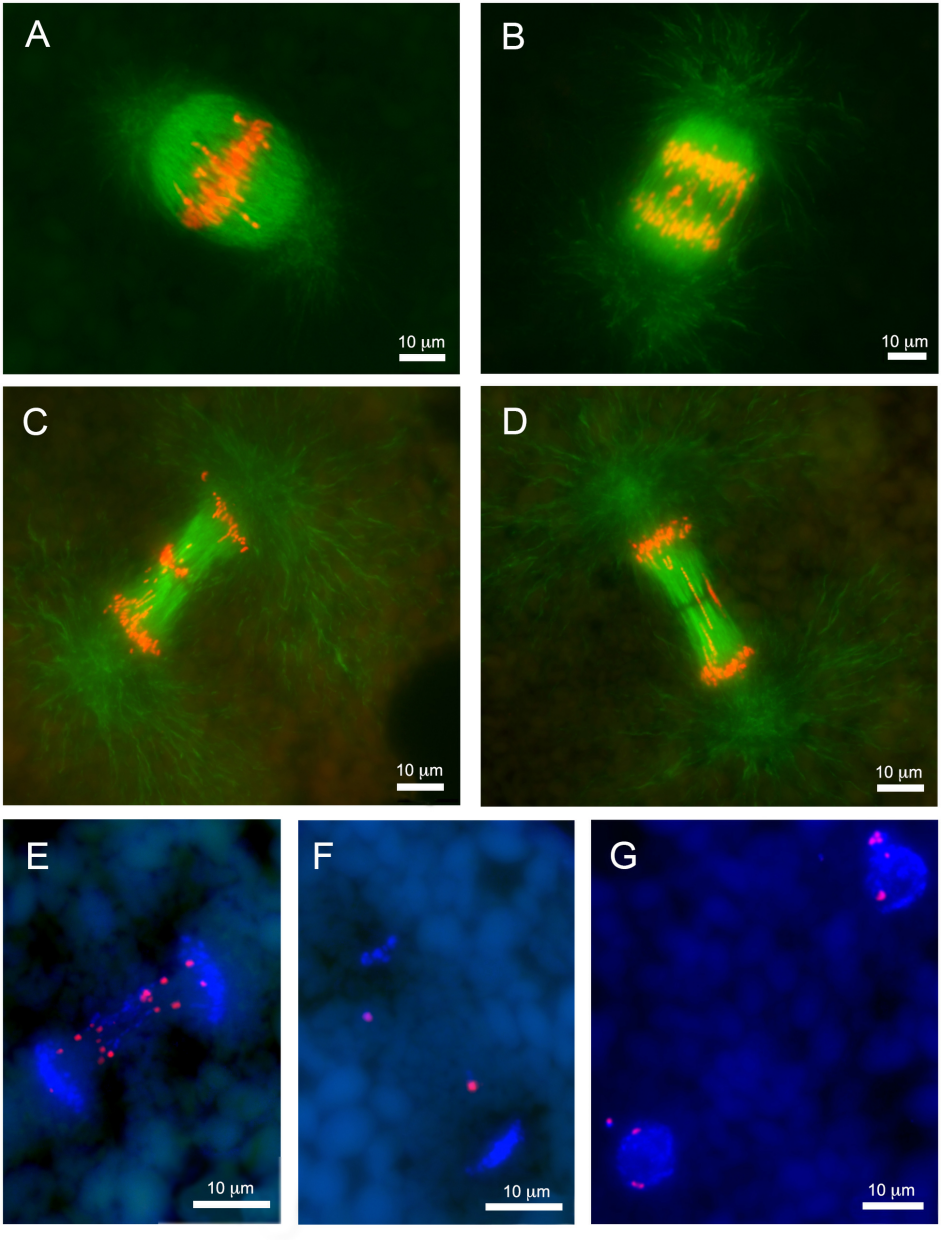
Lagging chromosomes are abundant during early stages of embryonic development. (A-D) Images of paraffin sections from lamprey embryos at 1 dpf. Anti-beta-tubulin immunolabeling: (A) metaphase, (B) anaphase A, (C) anaphase B with conglomerated chromatin in the equatorial area, (D) anaphase B with longitudinally stretched lagging chromatin. (E-F) Fluorescence *in situ* hybridization of the *Germ1* probe to embryo cells at 2 dpf from paraffin sections. (E) Anaphase with lagging chromosomes contain multiple signals for *Germ1*, somatic chromosomes retain a single pair of *Germ1* signals. (F) Late anaphase with two *Germ1*-positive micronuclei situated between condensing daughter nuclei. (G) Interphase cells with a single pair of *Germ1* signals in their main nuclei and additional signals in micronuclei.

Our in-depth analyses of MNi and their associated chromatin modifications revealed other cellular features that appear to be associated with PGR. The most striking among these were numerous anaphases with large amounts of lagging chromatin (Fig 2). Although these lagging anaphases were often visible in sections, the spindle apparatus often spanned more than 50 micrometers in rearranging embryos. As such, wax sections rarely permitted observation of entire anaphases (Fig 2F). To study detailed morphology of lagging anaphases we adapted the passive CLARITY technique (PACT) to whole lamprey embryos [35]. This approach increases the permeability of cells with minimal impact on the morphology of the embryos and effectively eliminates autofluorescence associated with yolk platelets (e.g. Figs 1 and 2). To complement this clearing method, we also optimized methods for DNA staining, fluorescence *in situ* hybridization, and β-tubulin immunolabeling of cleared lamprey embryos. Altogether, these analyses provide critical perspective on the developmental context of PGR and the dynamic behavior and packaging of eliminated DNA within rearranging cells.

**Fig 3:**
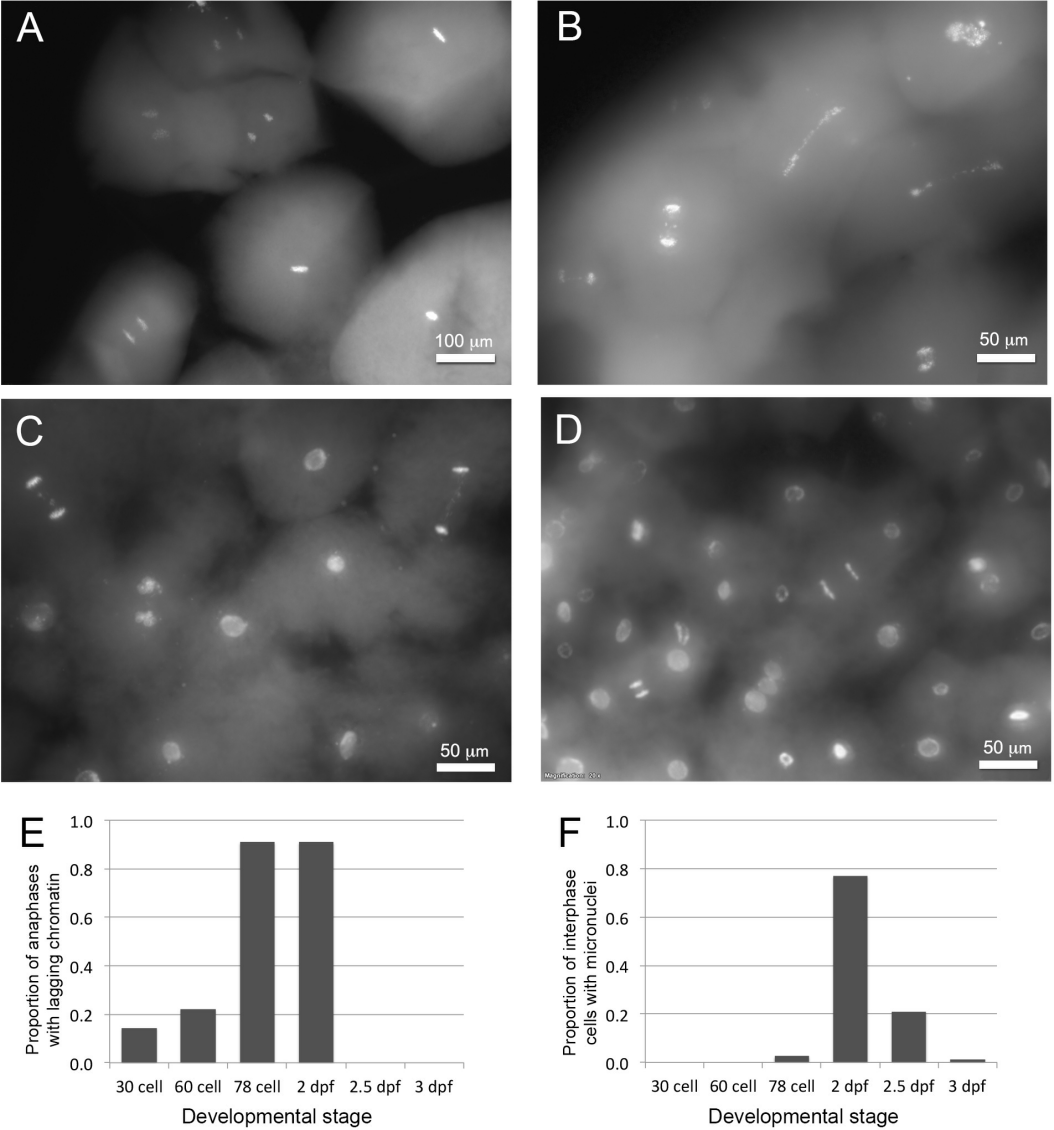
Timing of chromatin elimination. (A-D) PACT-cleared embryos, stained to highlight DNA (SYTO-24). (A) Cells from a 30-cell embryo at 1 dpf. Interphase cells lack micronuclei, and no lagging chromosomes are visible. (B) Cells from a 78-cell embryo at 1 dpf showing numerous anaphases with lagging chromatin. (C) Cells from an embryo showing anaphases with lagging chromatin and interphase cells with micronuclei. (D) Cells from an embryo at 2.5 dpf with few visible micronuclei and anaphases without lagging chromatin (presumably reflecting the completion of programmed genome rearrangement during earlier cell divisions). (E) Observed proportions of anaphases with lagging chromatin across early developmental stages. (F) Observed proportions of interphase cells containing micronuclei across early developmental stages.

We were able to establish a timeline for the onset and completion of PGR by examining PACT-cleared embryos across the first several days of development, leveraging natural variation in cell division rate during the first day post fertilization. Lagging anaphases were essentially absent during the first five to six cell divisions (e.g. in embryos with 30–60 cells) but abundant in embryos with more than 64 cells, suggesting PGR is initiated at approximately the onset of the seventh cell division (Fig 3). Lagging anaphases were also present at similarly high abundance at 2 dpf but dramatically decreased in frequency thereafter (S3 Table, S3A and S3B Fig). Notably, lagging chromosomes are observed earlier in development than MNi and peak in abundance at earlier developmental stages (Fig 3F; S3B and S3C Fig). We interpret the earlier appearance of lagging anaphases relative to MNi, as indicative of eliminated material initially slated for elimination during metaphase or early anaphase, and secondarily packaged into MNi.

### Behavior of Eliminated DNA through the Cell Cycle

Detailed examination of embryos at 1–3 dpf also revealed a graded series of cellular morphologies that appear to track the progression of DNA loss both within and between cell cycles. These morphological features provide additional perspective on the cellular and mechanistic details of elimination. Below we describe several salient features of eliminated chromatin, including its subcellular organization across the cell cycle and its association with cytoskeletal components.

**Fig 4:**
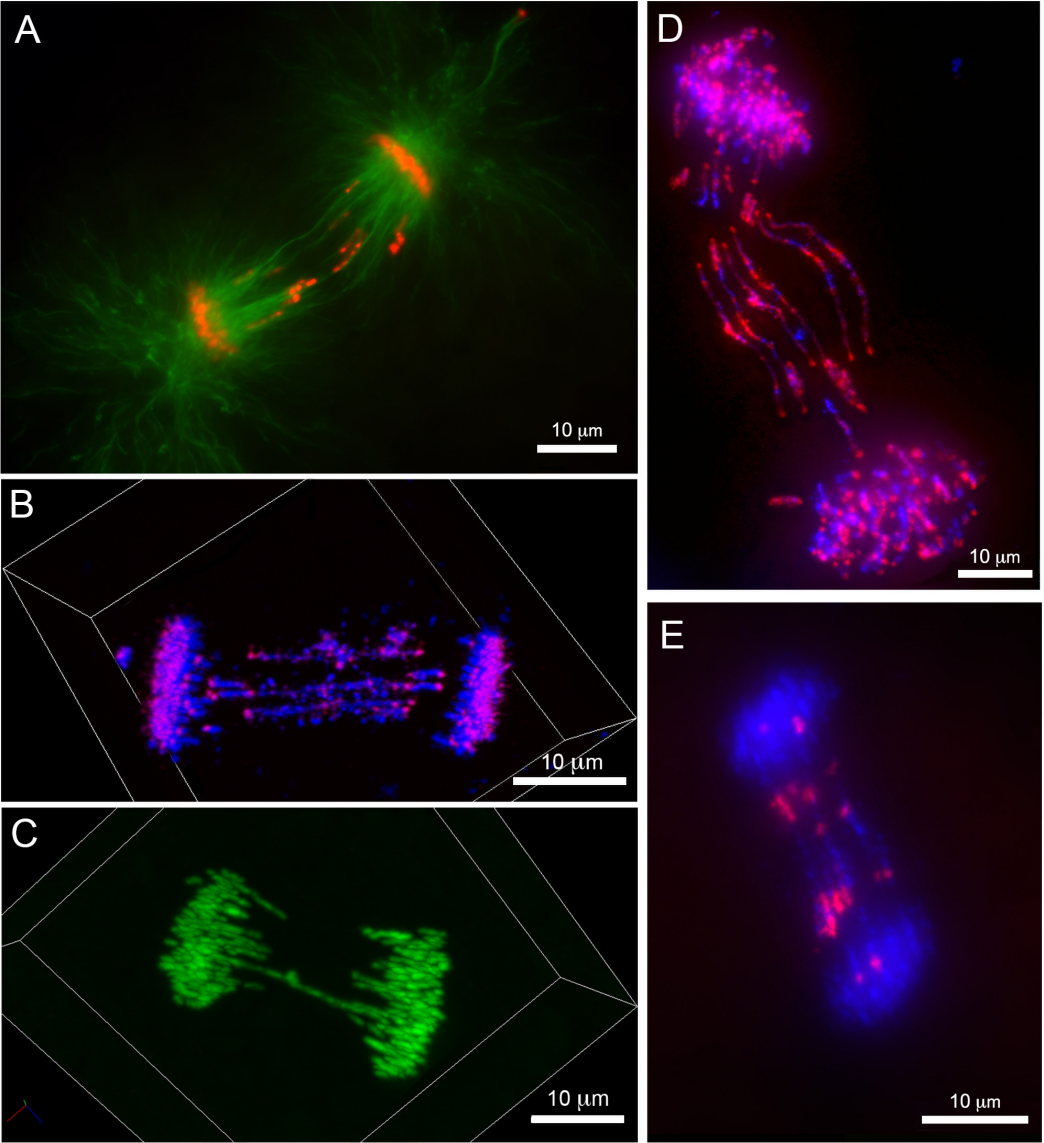
Morphology of anaphase lagging chromatin in intact embryonic cells. Immunolabeling and hybridization of intact, PACT-cleared, embryos (2 dpf). (A) immunolabeling with anti-beta-tubulin, lagging chromatin is oriented along the polar microtubules. (B) Confocal image of an anaphase labeled with a centromere specific probe (Cot1 FISH). Centromeres of lagging chromosomes are oriented toward the poles of mitotic spindle. (C) Confocal image of an anaphase from an embryo at 1dpf: lagging chromosomes form equatorial contacts, bridging between the poles of the mitotic spindle. (D) An anaphase with multiple bridging chromosomes, hybridized with a probe to repetitive DNA (Cot2 FISH). Punctate signals corresponding to centromeres are oriented toward the spindle poles but lag behind retained chromosomes. Sites of apparent contact between sister chromatids hybridize strongly to Cot2 DNA, suggesting that repetitive DNA may participate in establishing these contacts. (E) Fluorescence *in situ* hybridization of the *Germ1* probe to an anaphase with several bridging chromosomes. *Germ1* signals are symmetrical, further supporting the interpretation that bridging features consist of pairs of sister chromatids, though notably, *Germ1* signals do not appear to overlap with the zone of contact between sister chromatids.

Within a cell cycle, eliminated chromatin is first identifiable as thread-like structures that are situated between groups of poleward-oriented chromosomes immediately after the metaphase/anaphase transition. As anaphase progresses, eliminated material begins to exhibit distinguishable differences in its apparent motion relative to retained chromosomes. Lagging chromatin is typically oriented parallel to the interpolar microtubules and appeared to be tightly associated with spindle filaments (Fig 4A; S4 Fig). As cells enter telophase, retained sister chromatids begin to decondense and adopt lobate structures consistent with decondensation of somatic chromosomes and recruitment of nuclear envelope proteins. Notably, lagging chromatin does not appear to decondense at this same time and associates with tubulin prior to being packaged into compact MNi (S4 Fig and S5 Fig).

*In situ* hybridization with *Germ1* and other repetitive sequences (Cot1 and 2 DNA) revealed that lagging chromatin was distributed symmetrically across the metaphase plane. Hybridization with Cot1 DNA revealed that the polar ends of migrating (retained) chromosomes are enriched in highly repetitive DNA (S6 Fig), consistent with the interpretation that Cot1 DNA strongly hybridizes to centromeres, as has been observed for other species [36–38]. Notably, labeled Cot1 DNA also localized to the distal ends of some lagging fragments, suggesting that these segments contain active centromeres that are capable of engaging the kinetochore microtubules and (slower) poleward motion [36, 37] (Fig 4B, S1 Movie). Moreover, poleward-oriented regions of lagging chromatin are highly enriched in H3K9me3 (S1B Fig), which is considered a hallmark of constitutive pericentromeric heterochromatin [39, 40]. We interpret the symmetry of labeling and polar orientation of centromeric regions of lagging chromosomes as indicating that a substantial fraction of eliminated material was replicated in the previous cell cycle, packaged into sister chromatids at metaphase and drawn poleward at anaphase, albeit at a slower rate than somatically retained chromosomes.

Direct confocal imaging of fluorescently stained chromosomes (Fig 4C, S2 Movie) and *in situ* hybridization of Cot2 DNA (Fig 4D) revealed that the equatorial ends of symmetrically stretched sister chromatids often lay in close proximity to one another throughout anaphase. These apparent contacts between sister chromatids exhibit enhanced hybridization to Cot2 DNA, suggesting the possibility that an as-yet undefined class of repetitive sequences may contribute to PGR by anchoring sister chromatids to one another during anaphase (Fig 4D). Notably, *Germ1* is not present at these points of contact and is generally located in regions closer to the presumptive centromeres (Fig 4E). Taken together, these observations indicate that some of the eliminated material consists of entire chromosomes or large chromosomal segments and suggest that chromatin/chromatin (or DNA/DNA) contacts between telomeric segments of sister chromatids might contribute to the decelerated migration of these large eliminated fragments.

**Fig 5:**
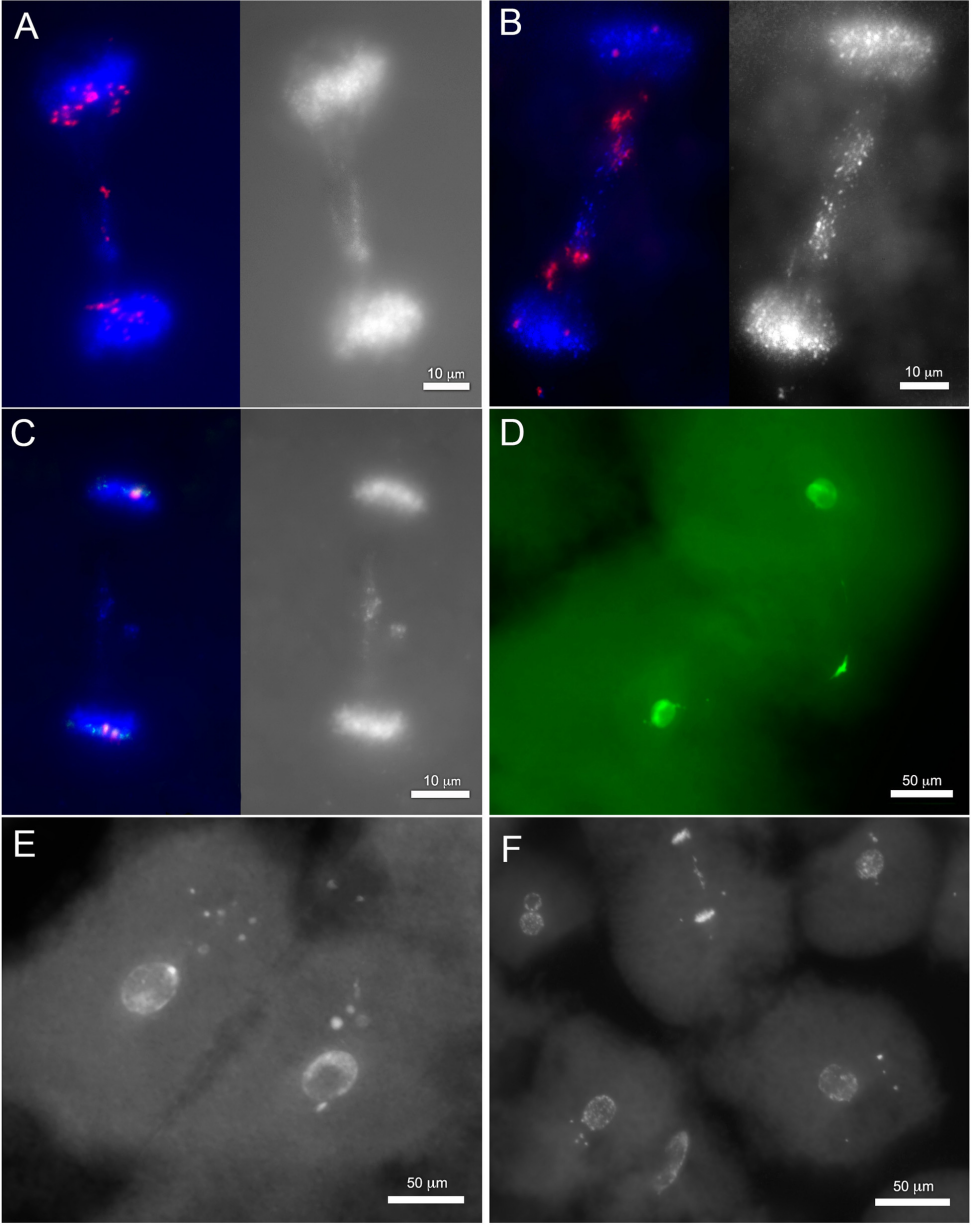
Variation in the content and form of eliminated DNA indicates stepwise loss of DNA. (A-C) FISH of the *Germ1* probe to anaphase chromosomes from PACT-cleared embryos. (A) A representative anaphase spread from 1 dpf (right panel: red—*Germ1*, blue—DAPI; left panel: DAPI). A majority of signals corresponding to the *Germ1* repeat co-migrate with retained chromosomes, and a relatively small conglomerate of chromatin is localized to the equatorial region. (B) A representative anaphase spread from 2 dpf (right panel: red—*Germ1*, blue—DAPI; left panel: DAPI). Lagging chromosomes are enriched with *Germ1* while retained chromosomes have only a single pair of *Germ1* signals. (C) *Germ1*-negative lagging chromatin at 2 dpf (right panel: red—*Germ1*, blue—DAPI; left panel: DAPI). *Germ1* hybridizes to a pair of signals on retained chromosomes that are associated with a relatively small conglomerate of lagging chromatin that lacks *Germ1* hybridization. (D-F) Late mitotic events in cells undergoing chromosome elimination. (D) Cytokinetic cells from 1 dpf possess dense and presumably heterochromatic structures located near the cleavage furrow with filamentous extensions oriented toward the enveloped nuclei (green—SYTO-24 stained DNA). Cells with multiple micronuclei from 1 dpf (E) and 2 dpf (F) stained with SYTO-24.

In addition to these large and longitudinally stretched segments, we also observed globular (presumable acentric) conglomerates of chromatin localized to the equatorial region (Fig 2C, see also Fig 5C). The presence of these conglomerates lends support to the idea that recombinational processes (intra- or inter-chromosomal) or DNA breakage contributes to PGR [4]. It seems plausible that these acentric fragments could be driven toward the equatorial region by the same polar ejection forces that normally act to orient chromosome arms during cell division [41]. The observation that eliminated material consists of both entire chromosomes and smaller chromosomal fragments mirrors observations from hagfish and parasitic nematodes, wherein both entire chromosomes and chromosomal fragments are lost from somatic lineages [2, 19, 42]. To shed further light on patterns of DNA breakage during PGR, we performed immunolabeling with an antibody to the histone variant γ-H2AX, which binds double stranded DNA breaks and recruits repair machinery [43, 44], and employed fluorescent TDT-mediated dUTP nick-end labeling (TUNEL) labeling to more generally detect DNA breaks. Although all other histone variants yielded interpretable signals, attempts to immunolabel γ-H2AX yielded no signal in embryos at 1–5 dpf. The absence of γ-H2AX immunolabeling could reflect either a paucity of double stranded breaks or failure to react with a lamprey γ-H2AX homolog. On the other hand, TUNEL labeling yielded strong and reproducible staining that was localized exclusively to MNi (S7 Fig). Given evidence that MNi represent the last visible sites of eliminated DNA, it seems plausible that TUNEL labeling reflects the degradation of germline-specific DNA within MNi. Taken together these observations indicate that DNA elimination proceeds through an ordered series of events, wherein germline-specific sequences 1) are initially slated for elimination during early anaphase (perhaps metaphase), 2) exhibit slower poleward movement in comparison to retained chromosomes and 3) condense to form MNi where they are methylated and ultimately degraded.

### Evidence for Progressive Elimination across Multiple Cell Cycles

Thus far, analyses of PGR in lamprey have revealed that patterns of gene loss are indistinguishable among diverse somatic cell lineages, which might be interpreted as supporting a simplistic model wherein all germline-specific sequences are eliminated during a single cell cycle [4, 6]. However, *in situ* hybridization of intact cells with *Germ1* appears to support a somewhat more complex model. As mentioned above, lamprey somatic cells possess a single pair of chromosomes that hybridize to the *Germ1* probe (S6A Fig), whereas *Germ1* hybridizes to several additional chromosomes in germ cells and embryonic cells that have not completed PGR [4]. As such, this marker can be used to track the progression of PGR. In early cell divisions (at 1 dpf) anaphases were observed that contained multiple *Germ1* signals interspersed among retained (normally migrating) chromosomes and relatively small amounts of lagging material, consistent with partial elimination of germline-specific sequences (Fig 5A). Variation in the process of elimination is also apparent in later developmental stages, as some anaphases possess two somatic *Germ1* signals and small amounts of *Germ1*-negative lagging chromatin (Fig 5B and 5C). These patterns suggest that cells had undergone at least one previous cycle of DNA elimination, over which they lost all germline-specific copies of *Germ1*, and were engaged in eliminating additional material at the time of fixation. The interpretation that PGR plays out over several cell cycles is further supported by the frequent observation of lagging chromatin and peripheral MNi within the same cell (S8 Fig). Presumably these peripheral MNi contain material that was eliminated in the previous cell cycle(s).

In this context, it is also worth noting that the earliest elimination events (1 dpf) appear to be associated with subcellular structures that are not observed at later stages. These appear as dense, presumably heterochromatic, structures located near the cleavage furrow with filamentous extensions oriented toward the enveloped nuclei (Fig 4D; S9 Fig). In general, these morphological features seem consistent with the interpretation that some (early) elimination events are characterized by persistent chromatin/chromatin or DNA/DNA contacts and that many of these same segments maintain an association with spindle microtubules. While it is possible that this variation is related to the fact that PGR is occurring in cells of vastly different sizes at 1 vs. 2 dpf (Fig 5E and 5F), it seems possible that the unique structures observed at 1 dpf might also reflect variation in the underlying mechanisms of PGR across early development.

### Broader Implications

Our analyses underscore the fact that evolution can arrive at diverse solutions to a common problem. Multicellular organisms employ a diversity of epigenetic silencing pathways, including covalent chemical modification of DNA or histones, expression of DNA binding factors (chromatin proteins and noncoding RNAs) that mediate the accessibility of DNA for transcription, and expression of short RNAs that promote degradation or prevent translation of transcripts. In general, these pathways are distributed broadly across diverse eukaryotic lineages, although individual pathways are evolutionarily labile [45–50], being retained in most lineages but absent from others. Programmed DNA elimination is sparsely distributed across the tree of life and likely arose several times over metazoan evolution [19], and in some cases PGR has been shown to act cooperatively with other silencing pathways (e.g. ciliates [11], sciarid flies [12, 13] and zebra finch [14]). It seems likely that each of these independent lineages has evolved its own approaches to achieve the reproducible elimination of DNA, prevent the loss of retained segments, and integrate these mechanisms with existing silencing pathways. As such, each of these lineages holds the potential to provide unique insights into a diversity of conserved (and derived) cellular mechanisms, including those that contribute to the proper segregation of chromosomes, epigenetic silencing, reconstitution of the nuclear envelope, and the maintenance of genome stability.

One notable feature of lamprey PGR is the variability in the content and form of eliminated chromatin across the first three days of development. Observations suggest that elimination events occurring ~1.5–2 dpf often target large regions (entire chromosomes) and appear to involve physical interactions between homologous chromosomes or sister chromatids. Earlier and later elimination events appear to target smaller fractions of the genome. The presence of variability across development raises several questions with respect to the mechanisms and outcomes of lamprey PGR. For example, does DNA loss involve a fixed number of steps/cell cycles? Do all elimination events share a common mechanism, or do new mechanisms/interactions arise later in development? Are later events uniform, or do they result in minor genetic variation across somatic cell lineages [6]? The variability observed over the time course of lamprey PGR is somewhat reminiscent of chromosome elimination in *Acricotopus lucidus* (Diptera, Chironomidae) [51]. In most cases, all germline-limited chromosomes are lost in a single mitosis, but rarely, one or several chromosomes escapes elimination and segregates with the somatically-retained chromosomes. Based on these observations, it has been suggested that a threshold exists wherein a certain number of hypothetical marks are necessary to drive elimination of *A*. *lucidus* chromosomes. As yet, it remains to be determined whether the observed variation apparent among lamprey elimination anaphases is programmatic, cell lineage specific, inherently noisy, or explained by threshold effects.

### Conclusions

The analyses presented here reveal several new cellular and molecular details related to developmentally programmed genome rearrangements in lamprey, a species that undergoes PGR in the context of a developmental and cellular biology that is largely conserved with other vertebrates [7–10]. Our analyses indicate that individual segments are slated for elimination during metaphase and are ultimately packaged into compact structures (micronuclei), a subset of which are enriched for repressive chromatin marks. These studies also demonstrate that PGR is initiated at an earlier developmental stage than was previously indicated via PCR-based assays [4] and strongly indicate that PGR is a more protracted process, being completed over the course of several successive cell divisions.

Based on our these new findings, we suggest that efforts to further dissect the mechanisms underlying lamprey PGR should include studies aimed at defining 1) the sequence of, and interactions between, repetitive sequences that occur in regions of contact between some eliminated chromosomes, 2) the role of epigenetic modifications (particularly silencing) in PGR and 3) interactions between eliminated DNA and components of the spindle apparatus/cytoskeleton. In addition to providing critical insights into the cellular and mechanistic basis of PGR, such studies are expected to aid in translating this information to systems wherein large-scale rearrangements and DNA losses are less programmatic and generally deleterious.

## Materials and Methods

### PACT

Clearing procedure was performed according to Yang et al. [35]. Paraformaldehyde fixed embryos were incubated in hydrogel monomer solution with 5% acrylamide supplemented 0.5% VA-044 overnight. Polymerization was performed at 37°C for 2.5 hours then embryos were washed briefly with PBS, and incubated in 8%SDS, 1x PBS for 5 days at 37°C with gentle shaking. FISH and immunolabeled samples were washed in 1x PBS with 5 buffer changes over the course of a day and transferred into staining solution (1x PBS, pH = 7.5, 0.1 Triton X-100, 0.01% sodium azide).

### Cytological Preparations

Spreads of somatic metaphase chromosomes were generated from embryos at 11 dpf. After overnight treatment with 0.1% colchicine, embryos were ground in Dounce homogenizer, incubated with 0.075 M KCl hypotonic solution for 45 minutes at room temperature and fixed in methanol:acetic acid (3:1). Cell suspensions were placed on glass slides and air-dried. Embryos for this and other experiments were produced under the University of Kentucky IACUC protocol number 2011–0848.

Paraffin sections were prepared for immunolabeling and FISH as follows. Sections were deparaffinized in two changes of xylene, gradually rehydrated in a dilution series of ethanol (100, 80, 70% in water), rinsed in water, and placed for overnight incubation in 10 mM sodium citrate buffer (pH 6.0) at 37^°^C to reduce auto-fluorescence and aid in antigen retrieval. Slides were then washed in PBS before hybridization and immunolabeling.

### DNA Probe Preparation and Fluorescence *In Situ* Hybridization (FISH)

Probes for *in situ* hybridization were labeled by nick-translation using direct fluorophores Cyanine 3-dUTP (Enzo Life Sciences, ENZ-42501) or Fluorescein-12-dUTP (Thermo Scientific, R0101) as described previously [52, 53]. *Germ1* repeat was obtained from a previously characterized BAC-clone [4] using extraction with Qiagen Large Construct kit (Qiagen Science, 12462). Cot1 and Cot2 fractions were isolated from genomic DNA according to kinetics of reassociation [54], using S1 nuclease to digest single stranded (low copy) DNA [38, 55]. Cot DNA isolation was performed in 1.2XSSC solution as follows: 120°C heating for shearing and denaturing, reannealing at 60°C, and S1 nuclease digestion for 1 hr at 42°C [55].

Whole embryo FISH was performed using modified procedure for cryosections [56]. Briefly, embryos were incubated in 10 mM sodium citrate buffer, pH = 6.0 overnight at 37°C in a rotating incubator, washed in 1x PBS for 1 hour, and then placed in 50% formamide in 2XSSC for 2–3 hours. For hybridization, formamide/SSC solution was replaced with 30 μl hybridization mix consisting of 50% formamide, 10% dextran sulfate, 0.01% sodium azide, and 150 ng labeled DNA-probe. Embryos were pre-incubated for overnight at 37°C to permit penetration of probes, after which probe and target DNA was denatured by heating samples to 75°C for 3 minutes. Following overnight incubation at 37°C samples were washed in 50% formamide in 2XSSC and in 0.4XSSC, 0.3% IGEPAL^®^ CA-630 (Sigma Cat. no. I8896) at 45°C for 10 min each, then in 2XSSC, 0.1% IGEPAL for 10 min at room temperature. DAPI and SYTO-24 counterstain was performed in staining solution at room temperature for, at least, 1 hour for embryos at 2–3 dpf and overnight for 1 dpf.

Fluorescence *in situ* hybridization of embryonic sections and mitotic spreads was carried out according to standard protocols [56, 57] with minute modifications [52]. Deparaffinized section slides were incubated in 8% sodium thiocyanate solution overnight, pretreated with 10 μg/ml RNase and 0.01% pepsin solutions, denatured in 70% formamide with subsequent dehydration in ethanol series (70, 80, 100%) and hybridized with 100–200 ng of probe overnight in humid chamber at 37°C. For chromosome spreads prehybridization treatments with sodium thiocyanate, RNase, and pepsin solutions were skipped.

### Immunolabeling

Primary antibodies for immunolabeling were as follows: monoclonal anti-5-Methylcytosine (Epigentek, A-1014), polyclonal anti-Histone H3-K9 Trimethyl (Epigentek, A-4036), polyclonal anti-Histone H3-K27 Trimethyl (Epigentek, A-4039), monoclonal anti-Beta Tubulin (Abcam, ab179513), polyclonal anti-Lamin B1 (Boster, PB9611) and monoclonal anti-Nuclear Pore-O-linked Glycoprotein (Thermo, MA1-071). Primary antibodies were diluted 1:100 in 1x PBS, applied to slides, and incubated overnight at 4^°^C. After washing in PBS and PBST twice for 10 min each, slides were incubated with secondary antibodies to their respective host species at a 1:100 dilution using the following antibodies: Alexa Fluor 488 F(ab')2 fragment of rabbit anti-mouse (LifeTechnologies, A-21204), Alexa Fluor 594 F(ab’)2 fragment of goat anti-mouse, Alexa Fluor 488 chicken anti-rabbit (LifeTechnologies, A-21441). After washing as described in PBS and PBST solutions, slides were mounted using VectaShield-DAPI media (Vector Laboratories, H-1400). Whole embryo immunofluorescence labeling was carried out according to methods previously described for single cell phenotyping [35]. Briefly, PACT-cleared embryos were incubated with primary antibodies (1:100, in PBS containing 10% normal serum of secondary antibody host species (rabbit), 0.1% Triton X-100 and 0.01% sodium azide) for 3 days, replacing antibodies daily. Unbound antibody was removed via PBS washes, and samples were incubated with secondary antibodies (1:100) for 2–3 days then washed for 1 day in PBS prior to incubation with DAPI (50 ng/ml) and imaging media (RIMS: 88% Histodenz (Sigma, D2158) in PBS with 0.1% tween-20 and 0.01% sodium azide, pH to 7.5). All staining and mounting steps were conducted at room temperature with gentle shaking.

### TUNEL Assay

TUNEL reactions were performed on paraffin sections from 2 dpf embryos. Slides were deparaffinized, treated with sodium citrate solution as above, and labeling was performed using the Click-iT Plus TUNEL Assay (Life Technologies Cat. no. C10617), according to manufacturer instructions. Samples were permeabilized with proteinase K for 30 min at 37°C. A positive control was generated by treatment with a 1:50 dilution of DNAseI (ThermoFisher Scientific Cat. no. EN0525) in reaction buffer, followed by incubation at room temperature for 30 min. TUNEL assays were performed on experimental and positive control slides simultaneously, then slides were mounted with VectaShield-DAPI media (Vector Laboratories, H-1400).

### Microscopy and Image Analysis

After FISH and immunolabeling, slides were analyzed with an Olympus-BX53 microscope using filter sets for DAPI, TexasRed, and FITC. Images were captured using CellSence software. For thicker samples, such as sections and embryonic cells after PACT clearing, we used Extended Focal Imaging (EFI) function in order to generate a single deep-focus image. Three-dimensional images of anaphases were obtained using a scanning confocal microscope (Nikon C2) equipped with NIS-Elements AR software. Three-dimensional images were converted in two-dimensional format in NIS Element Viewer. Pseudocolor corrections were performed using Adobe Photoshop CS6. Video recordings were made in NIS Element Viewer using QuickTime media player “Screen recording” function.

### Statistical Analyses

The frequency of MNi in paraffin sections was assessed by counting DAPI-stained primary nuclei and small extra-nuclear DAPI-positive structures. For FISH and immunolabeling experiments, MNi were counted as signal-positive when they yielded visible DAPI emission and fluorescence in the specific wavelength corresponding to the fluorophore used for detection. Between 20 and ~200 primary nuclei were counted per slide, depending on stage of development. Fewer nuclei were counted for earlier stages due to the fact that these embryos consist of smaller numbers of larger cells. Counts of MNi, anaphases and lagging anaphases were performed after hybridization of whole embryos with fluorescently-labeled Cot2 DNA in order to improve visualization of eliminated DNA. Frequencies of MNi, anaphases and lagging anaphases were compared between adjacent time points using Pearson’s chi-square test and by calculation of Bayesian central confidence intervals [58].

## Supporting Information

S1 Fig H3K9me3/5MeC immunolabeling of lamprey cells. (**A-C**) Cells of 2 dpf embryos at (**A**) metaphase, (**B**) anaphase and (**C**) after cytokinesis. (**D**) Cells of a 3 dpf embryo at interphase. (**E**) Cells of a 7 dpf embryo at interphase. For all panels, anti-H3K9me3 immunofluorescence is shown in green, 5MeC in red, and DNA (DAPI counterstain) in blue. Positively-labeled and signal-negative micronuclei are marked by stemmed and notched arrows, respectively.
(TIF)

S2 Fig Immunolabeling of nuclear envelope markers in rearranging embryos. **(A-B)** Images of paraffin sections from lamprey embryos at 1.5 dpf, immunolabeled with **(A)** anti-Lamin-B1 and **(B)** anti-nuclear pore o-linked glycoprotein. Both of these proteins localize to interphase nuclei, but are absent from micronuclei. For merged images, immunofluorescence is shown in green and DNA (DAPI counterstain) is shown in blue.
(TIF)

S3 Fig Proportion of cells in anaphase stage and interphase cells with MNi in PACT-cleared whole embryos. Histograms show changes in the **(A)** anaphase index, **(B)** proportion of anaphases with lagging chromatin, and **(C)** formation of micronuclei, over the first three days of lamprey embryogenesis.
(TIF)

S4 Fig Tracing lagging chromatin from late anaphase through telophase. PACT-cleared embryos at 2 dpf showing the localization of chromosomes (red) and beta-tubulin (green) or chromosomes (greyscale). (**A, B**) late anaphase, (**C-F**) telophase/cytokinesis with karyomeres in the process of merging into daughter nuclei. Lagging chromosomes lie parallel to the longitudinal axis of polar spindle fibers while retained chromosomes are packaged into interphase nuclei.
(TIF)

S5 Fig Progressive formation of micronuclei and delay of envelope assembly around lagging chromatin. (**A**) Telophase cells from PACT-cleared embryos. Thread-like lagging chromatin extends between assembling nuclei. (**B-C**) Cells in late telophase and after cytokinesis. Retained chromatin has aggregated into rounded structures consistent with recruitment of nuclear membrane, lagging chromatin has begun to be packaged into discreet rounded structures (micronuclei), which are physically separated from the main nuclei. (**D**) Cell in telophase (karyomere assembly) from a 2 dpf embryo (paraffin section). Lagging chromatin has not coalesced into rounded vesicles indicating a delay in nuclear envelope assembly. A-C: SYTO DNA-staining in whole PACT-cleared embryos. D: DAPI staining.
(TIF)

S6 Fig Fluorescence *in situ* hybridization Cot1 repetitive DNA (red) on lamprey chromosomes. (**A**) A metaphase spread of a somatic cell from an 11-dpf embryo. Signals corresponding to Cot1 repetitive fraction are localized primary in pericentromeric regions of all acrocentric chromosomes of lamprey. Green signals correspond to a single pair of *Germ1*-carrying chromosomes. (**B**) Confocal image of an anaphase from a 2 dpf embryo. Poleward orientation of dot-like signals is consistent with the interpretation that these Cot1-hybridizing regions correspond to pericentromeric repeats.
(TIF)

S7 Fig Detection of DNA breaks in paraffin section of 2 dpf lamprey embryos (TUNEL assay). **(A)** Individual DAPI/FITC channels and a merged image of interphase cells, with micronuclei demonstrating strong fluorescence of labeled DNA breaks. **(B)** A positive labeling control that has been pre-treated with DNAseI, showing bright fluorescence of primary nuclei. For merged images, FITC is shown in green and DNA (DAPI counterstain) is shown in blue.
(TIF)

S8 Fig Examples of anaphases with lagging chromosomes and peripheral micronucleated chromatin. (**A, B**) Anaphases that have been hybridized with a fluorescent probe for the *Germ1* repeat. (**C, D**) Anaphases that have been hybridized with labeled Cot2 DNA (repetitive DNA). (**E**) Anaphase that have been hybridized with labeled Cot1 DNA (higher copy releats). Arrows mark micronucleated chromatin. The localization of micronuclei external to the polar microtubules suggests that these micronuclei are derived from lagging chromatin that was excluded from the nucleus in the previous cell cycle(s). FISH-signals are shown in red, DNA is counterstained with DAPI (blue).
(TIF)

S9 Fig Examples of cytokinetic morphology 1^st^ dpf embryos with specific chromatin structures extending between daughter nuclei, across the cleave plane. PACT-cleared embryos stained with SYTO-24.
(TIF)

S1 Movie Three-dimensional video-image of anaphase from 1 dpf embryo after hybridization with Cot1 DNA (red) and staining with SYTO-24 (green). Stretched lagging chromosomes oriented antiparallel from pole to pole with poleward-oriented centromeric regions.
(MOV)

S2 Movie Three-dimensional video-image of anaphase from a 1 dpf embryo. Lagging chromatin stretched from pole to pole forms apparent contact in the spindle equatorial region. DNA has not undergone denaturation or hybridization. DNA is stained with SYTO-24 (green).
(MOV)

S1 Table *In situ* hybridization of the germline-specific marker Germ1. Proportions of micronuclei showing Germ1 hybridization during lamprey embryogenesis.
(PDF)

S2 Table Micronuclei and epigenetic modifications. Changes in abundance of micronuclei and epigenetic modifications during the first week of lamprey development.
(PDF)

S3 Table Lagging chromatin in the context of early embryogenesis and PGR. Proportions of cells in anaphase, with lagging anaphases and with micronuclei, over the first three days of lamprey development.
(PDF)
