# Supplementary figures and images for: Cellular and Molecular Features of Developmentally Programmed Genome Rearrangement in a Vertebrate (Sea Lamprey: Petromyzon marinus)
Source: PLoS Genet. 2016 Jun 24;12(6):e1006103. doi: 10.1371/journal.pgen.1006103 (PMC4920378; doi:10.1371/journal.pgen.1006103)

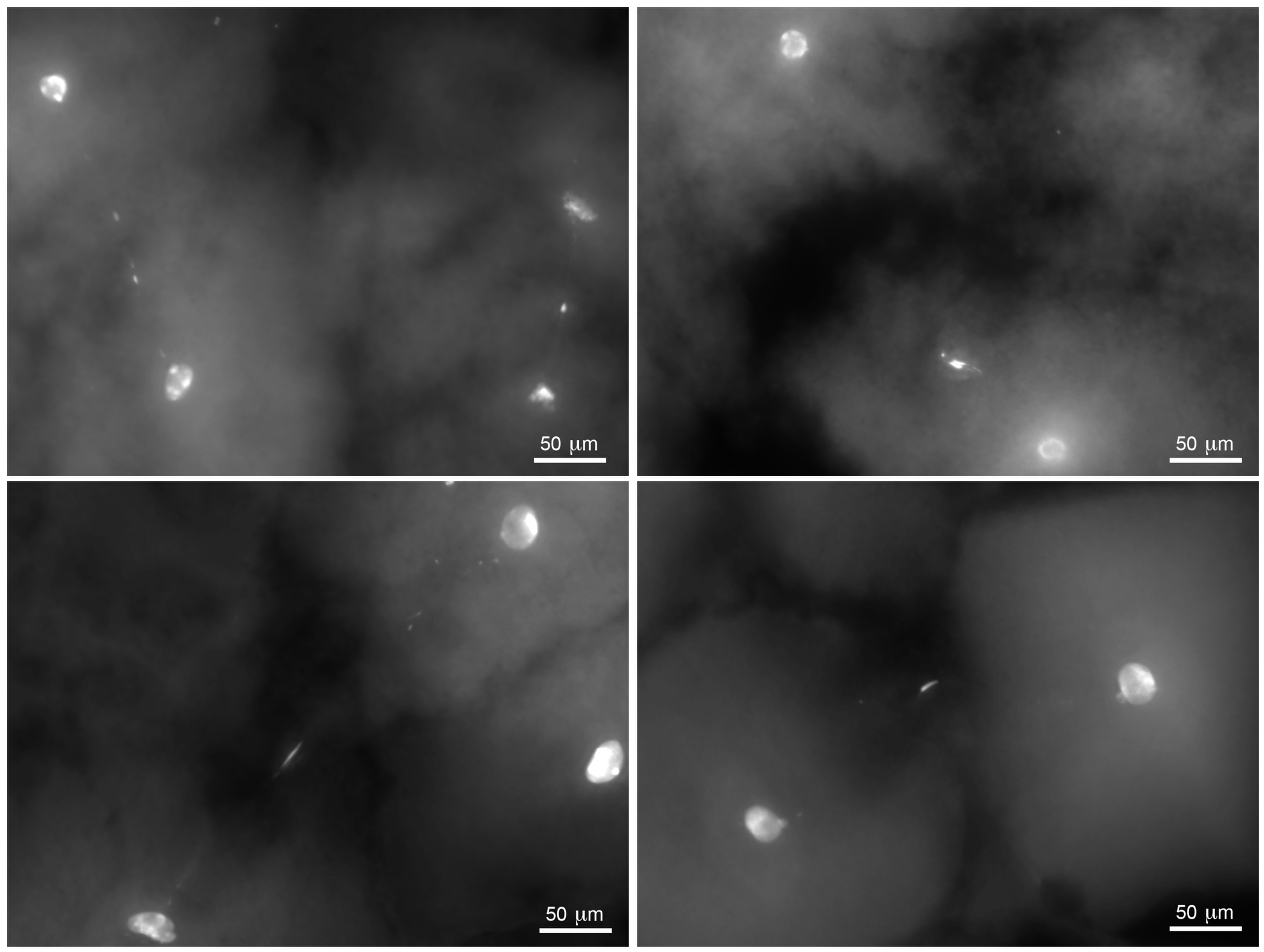

Supplement: S9 Fig — PACT-cleared embryos stained with SYTO-24. (TIF) [file pgen.1006103.s009.tif]
