# Supplementary material for: Cellular and Molecular Features of Developmentally Programmed Genome Rearrangement in a Vertebrate (Sea Lamprey: Petromyzon marinus)
Source: PLoS Genet. 2016 Jun 24;12(6):e1006103. doi: 10.1371/journal.pgen.1006103 (PMC4920378; doi:10.1371/journal.pgen.1006103)
Supplement: S1 Table — Proportions of micronuclei showing Germ1 hybridization during lamprey embryogenesis. (PDF) [file pgen.1006103.s012.pdf]

S1 Table

***In situ* hybridization of the germline-specific marker *Germ1*.** Proportions of micronuclei showing *Germ1* hybridization during lamprey embryogenesis.

Days post fertilization	Number of nuclei	Micronuclei		Proportion of *Germ1*+ MNi (95% Bayesian central confidence interval)	Change in proportion of MNi with *Germ1* hybridization relative to previous time point
		Total	*Germ1*+		
1	21	4	4	**1** (0.48-0.99)	-
1.5	139	150	133	**0.887** (0.83-0.93)	-0.11
2	111	118	88	**0.746** (0.66-0.82)	-0.14**
2.5	152	94	71	**0.755** (0.66-0.83)	0.01
3	155	55	48	**0.873** (0.76-0.94)	0.12
5	202	16	12	**0.750** (0.50-0.90)	-0.12
7	200	0	0	**-**	-

** Pearson's χ^2 $p<0.01$
