# Supplementary material for: Cellular and Molecular Features of Developmentally Programmed Genome Rearrangement in a Vertebrate (Sea Lamprey: Petromyzon marinus)
Source: PLoS Genet. 2016 Jun 24;12(6):e1006103. doi: 10.1371/journal.pgen.1006103 (PMC4920378; doi:10.1371/journal.pgen.1006103)
Supplement: S2 Table — Changes in abundance of micronuclei and epigenetic modifications during the first week of lamprey development. (PDF) [file pgen.1006103.s013.pdf]

S2 Table

Micronuclei and Epigenetic Modifications. Changes in abundance of micronuclei and epigenetic modifications during the first week of lamprey development.

Days post fertilization	Number of primary nuclei	Number of micronuclei				Change in MNi abundance relative to previous time point	Proportion of MNi 5MeC+		Proportion of MNi H3K9me3+	
		Total	5MeC+	H3K9 me3+	5MeC+ H3K9 me3+		(95% Bayesian central confidence interval (CI))	Change from previous	(95% CI)	Change from previous
1	18	8	0	5	0	-	**0.00** (0.00-0.34)	-	**0.63** (0.30-0.86)	-
1.5	70	101	7	57	0	0.19**	**0.07** (0.03-0.14)	0.07	**0.57** (0.47-0.66)	-0.06
2	103	155	96	2	0	0.00	**0.62** (0.54-0.69)	0.55**	**0.01** (0.004-0.05)	-0.55**
2.5	118	114	62	4	0	-0.05	**0.54** (0.45-0.63)	-0.08	**0.04** (0.01-0.09)	0.02
3	191	146	48	5	1	-0.22**	**0.33** (0.26-0.41)	-0.22**	**0.03** (0.02-0.08)	0.00
5	134	71	3	2	2	-0.29**	**0.04** (0.02-0.12)	-0.29**	**0.03** (0.01-0.10)	-0.01
7	209	23	0	0	0	-0.07	**0.00** (0.00-0.14)	-0.04	**0.00** (0.00-0.14)	-0.03

** Pearson's χ^2 $p<0.01$
