# Supplementary material for: Cellular and Molecular Features of Developmentally Programmed Genome Rearrangement in a Vertebrate (Sea Lamprey: Petromyzon marinus)
Source: PLoS Genet. 2016 Jun 24;12(6):e1006103. doi: 10.1371/journal.pgen.1006103 (PMC4920378; doi:10.1371/journal.pgen.1006103)
Supplement: S3 Table — Proportions of cells in anaphase, with lagging anaphases and with micronuclei, over the first three days of lamprey development. (PDF) [file pgen.1006103.s014.pdf]

S3 Table

Lagging chromatin in the context of early embryogenesis and PGR. Proportions of cells in anaphase, with lagging anaphases and with micronuclei, over the first three days of lamprey development.

Days post fertilization	Number of interphase cells	Number of anaphases	Number of lagging anaphases	Number of MN+ cells	Anaphases				Proportion of interphase cells with MNi	
					Proportion of cells		Proportion lagging			
					(95% Bayesian central confidence interval (CI))	Change from previous	(95% CI)	Change from previous	(95% CI)	Change from previous
1	98	21	12	11	**0.18** (0.12-0.25)	-	**0.57** (0.36-0.76)	-	**0.11** (0.06-0.19)	-
2	137	33	30	121	**0.19** (0.14-0.26)	0.02	**0.91** (0.76-0.97)	0.34**	**0.88** (0.82-0.93)	0.77**
2.5	198	24	0	56	**0.11** (0.07-0.16)	-0.09*	**0.00** (0.00-0.14)	-0.91**	**0.28** (0.22-0.35)	-0.60**
3	225	2	0	2	**0.01** (0.003-0.03)	-0.10**	**0.00** (0.00-0.70)	-	**0.01** (0.003-0.03)	-0.27**

* Pearson's χ^2 $p<0.05$, ** Pearson's χ^2 $p<0.01$
